# Remarkable impact of low BiYbO_3_ doping levels on the local structure and phase transitions of BaTiO_3_†
†Electronic supplementary information (ESI) available: Refined coordinates, temperature factors (*U*_iso_) and bond lengths, SEM, P–E and S–E measurements. See DOI: 10.1039/c7ta11096k


**DOI:** 10.1039/c7ta11096k

**Published:** 2018-03-15

**Authors:** M. Deluca, Z. G. Al-Jlaihawi, K. Reichmann, A. M. T. Bell, A. Feteira

**Affiliations:** a Materials Center Leoben Forschung GmbH , Roseggerstrasse 12 , 8700 Leoben , Austria . Email: marco.deluca@mcl.at; b Institut für Struktur-und Funktionskeramik , Montanuniversitaet Leoben , Peter Tunner Strasse 5 , 8700 Leoben , Austria; c Materials and Engineering Research Institute , Sheffield Hallam University , S1 1WB , Sheffield , UK . Email: a.feteira@shu.ac.uk; d Institute for Chemistry and Technology of Materials , Graz University of Technology Stremayrgasse 9 , 8010 Graz , Austria

## Abstract

*In situ* Raman spectroscopy shows the simultaneous incorporation of small amounts of Bi^3+^ and Yb^3+^ into the lattice of BaTiO_3_ to break the average symmetry inferred from X-ray powder diffraction analysis and permittivity measurements. In particular, Bi^3+^ with a stereochemically active lone-pair of electrons induces severe lattice strain and the coexistence of different local crystal symmetries over a wide temperature range, effectively controlling the physical properties, such as the temperature dependence of the permittivity and the Curie temperature. These results show that compositional gradients based in small variations of these two dopants could successfully explain the enhanced thermal stability of the permittivity in core–shell type ceramics, whereas the lower capacitance of the shell can also cap the maximum permittivity at the Curie temperature.

## Abstract 2

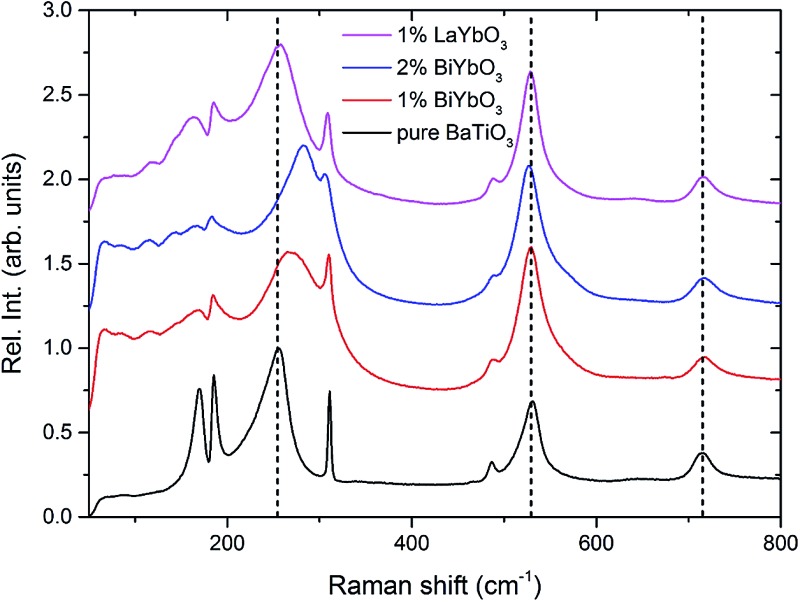
Bi^3+^ with a stereochemically active lone-pair of electrons induces severe lattice strain in BaTiO_3_ as revealed by a significant Raman shift of the mode associated with the O–Ti–O bonds.

## Introduction

Recent research into energy storage, lead-free piezoelectrics and high temperature stable dielectrics has renewed the interest on BaTiO_3_-solid solutions incorporating Bi^3+^. In particular, there has been considerable interest in the dielectric and piezoelectric properties of BaTiO_3_–BiMO_3_ (M = Al, Sc, Fe, In, Y, Gd, Yb, …)^1^–^5^ ceramics, but also on BaTiO_3_–Bi(M_B1_M_B2_)O_3_ (M_B1_M_B2_ = Zn_1/2_Ti_1/2_, Zn_1/2_Zr_1/2_, Mg_1/2_Ti_1/2_, Mg_2/3_Nb_1/3_, …).^6^–^8^ Interestingly, these solid solutions appear to exhibit some peculiar dielectric phenomena, such as a weakly-coupled relaxor behaviour, which is remarkably stable up to high temperatures and high electric fields. The temperature- and frequency-dependent dielectric response can be simultaneously described by the Curie–Weiss law and the polar nanoregions (PNRs) model applied to traditional relaxors. Nevertheless, Vogel-Fulcher analysis revealed activation energies one order of magnitude higher that those observed for traditional relaxors. Some models have been proposed to explain the aforementioned dielectric characteristics. For example, the BaTiO_3_–BiAlO_3_ and BaTiO_3_–BiScO_3_ systems have been reported to show a so-called “re-entrant like relaxor behaviour”,^9^ which manifests itself by the appearance of relaxor-type behaviour at temperatures above the permittivity maximum. Recently, a double rattling ion model^1^ was proposed to describe the temperature- and frequency-dependence of the polarizability in BaTiO_3_–BiMO_3_ materials. Basically, the larger Ba^2+^ and M^3+^ ions create oversized A-site cages for Bi^3+^ions, resulting in off-centering of Bi^3+^ in order to create shorter Bi–O bonds and thereby relieve the tensile bond strain. Hence, both Bi^3+^ and Ti^4+^ prefer displaced positions, and it is the dynamic hopping among these positions that gives rise to the dielectric relaxation. This relaxation is often observed at relatively high BiMO_3_ contents. At lower BiMO_3_ contents other phenomena can be observed. For example, the BaTiO_3_–BiYbO_3_ system also shows a so-called “ferroelectric-to-relaxor crossover”^3^ at *x* = ∼0.05, where the emergence of relaxor-type behaviour occurs in compositions, whose average crystal symmetry is still described by the *P*4*mm* tetragonal space group.

The average long-range structure of BaTiO_3_ is well understood. On cooling, the crystal symmetry of BaTiO_3_ changes successively from cubic (*Pm*3*m*) to tetragonal (*P*4*mm*) at ∼130 °C, then to orthorhombic (*Amm*2) at 5 °C and finally to rhombohedral (*R*3*m*) at –90 °C. These phase transitions are accompanied by discontinuities in relative permittivity, unit cell volume and frequency of some lattice vibration modes. Indeed, the fundamental properties of BaTiO_3_ are related to phonons of the optical branch, in particular the paraelectric-to-ferroelectric phase transition appears connected to the softening of the lowest frequency transverse optical (TO) mode. The static dielectric constant and the structural phase transitions of BaTiO_3_ are also related to phonons of optical frequency. Hence, Raman spectroscopy is often employed to investigate the impact of chemical doping on the lattice dynamics and consequently infer the dielectric behaviour. Pokorny *et al.*^10^ demonstrated that site occupancy in doped BaTiO_3_ can be analysed by Raman spectroscopy, in particular the appearance of an A_1g_ mode was postulated to be a sufficient proof to ascertain either the presence of a dopant in the B-site or the existence of a Ti vacancy. In Bi-substituted BaTiO_3_, Deluca *et al.*^11^ proved that the insertion of Bi^3+^ into the BaTiO_3_ lattice promotes tetragonal distortion due to the effect of the stereochemically active lone-pair of Bi^3+^. This effect, however, seems to be compensated if a large cation (such as Yb) is introduced on the B-site.^2^ These aspects were probed by Raman spectroscopy. Studies of the impact of low BiMO_3_ doping contents on the dielectric and vibrational characteristics of BaTiO_3_–BiMO_3_ ceramics are seldom, however they may provide a further insight on the peculiar properties of these systems.

Moreover, minute amounts of trivalent rare-earth (RE^3+^) are commonly used as dopants in commercial formulations of multilayer ceramic capacitors to improve their reliability. It was found that the electrical properties of A-site RE^3+^ doped BT are very dependent on the processing conditions, such as the oxygen partial pressure, sintering temperature and rate of cooling from the sintering temperature.^12^ For example, it was shown that La-doping in BT ceramics fired in air leads to the formation of Ti-vacancies, but the emergence of semiconductivity results from oxygen loss, which appears more favourable in the doped system than in undoped BT.^12^ It has been also shown that semiconductivity in acceptor doped BaTi_1–*x*_Ho_*x*_O_3_ ceramics with *x* = 0.001 to 0.01 is due to the presence of Ti^3+^. It was suggested that for these diluted Ho contents, each oxygen vacancy is charge compensated by one Ho^3+^ and one Ti^3+^, whereas at higher *x* values charge compensation is achieved by Ho^3+^ and the samples are insulating.^13^

Here, the dielectric behaviour of charge compensated (1–*x*)BaTiO_3_–*x*BiYbO_3_ (0 ≤ *x* ≤ 0.02) ceramics was investigated using a combination of temperature-dependent dielectric measurements and *in situ* Raman spectroscopy. In agreement with the dielectric measurements, Raman spectroscopy data show the incorporation of Bi/Yb to have little impact on the cubic-to-tetragonal structural phase transition but to affect dramatically the tetragonal-to-orthorhombic transition. Long-range covalence effects brought by Bi^3+^ in the (1 – x)BaTiO_3_–*x*BiYbO_3_ system, are evidenced through the comparison with the behaviour of a control sample (1 – *y*)BaTiO_3_–*y*LaYbO_3_ with *y* = 0.01, where La^3+^ does not feature such a lone-pair of electrons. Bi^3+^ with an ionic radius of 1.45 Å exhibits a 6s^2^6p^0^ valence electronic configuration, where the 6s^2^ lone-pair of electrons hybridize with empty 6p^0^ orbitals of Bi^3+^ but also with the 2p^6^ electrons of O^2–^ forming mainly covalent Bi–O bonds, leading to local structural distortion. La^3+^ with an ionic radii of 1.36 Å exhibits 4d^10^5s^2^5p^6^ and may also form covalent bonds. Nevertheless, based on the electronegativity difference it can be anticipated that La–O bonds should exhibit a larger ionic character than the Bi–O bonds. Hence, (1 – *y*)BaTiO_3_–*y*LaYbO_3_ ceramics with *y* = 0.01 were prepared in order to evaluate the impact of local bond character on the long-range structure. For higher LaYbO_3_ contents the reader is refer to a previous study by Feteira and Sinclair,^14^ which shows the coalescence of all structural phase transitions at *y* = 0.05.

## Experimental

Dried BaCO_3_ (>99.0%), TiO_2_ (>99.9%, Aldrich), Bi_2_O_3_ (>99.9%, Aldrich), Yb_2_O_3_ (>99.9%, Aldrich) powders were weighed according to the Ba_1–*x*_Bi_*x*_Ti_1–*x*_Yb_*x*_O_3_ formula for *x* = 0, 0.005, 0.01 and 0.02. These powders were placed into a 250 ml milling polyethylene bottle together with ∼0.5 kg of yttrium-stabilized zirconia milling media and ∼100 ml of propan-2-ol and then mixed on roller ball mill overnight. Mixed powders were dried and then passed through a 500 μm mesh sieve. The sieved powders were pressed into pellets and reacted between 850 and 1000 °C with intermittent re-grinding and re-firing until no change on X-ray diffraction (XRD) data was visible. The fully reacted powders were pressed as 8 mm pellets and fired up to 1300 °C for 2 hours. The pellets were stacked on top of each other and sintered in a closed alumina crucible to limit loss of Bi. Their microstructures (given in the ESI†) were examined by scanning electron microscopy using a SEM equipped with a W-filament. All doped ceramics exhibited similar grain sizes and morphologies. In addition, Ba_1–*y*_La_*y*_Ti_1–*y*_Yb_*y*_O_3_ with *y* = 0.01 was prepared as a control sample, in order to contrast the impact of Bi^3+^ and La^3+^. Purity and crystal structure analyses were carried out by XRD using a Bruker diffractometer (model D8) set up in transmission geometry and using monochromatic Cu Kα_1_ radiation. XRD patterns were acquired in the 20–70 2*θ* range, with a step size of 0.02 degrees with a scan length of 2 seconds per step. Rietveld refinements were done using FULLPROF.^15^ Raman spectra were obtained with a Renishaw Raman microscope (model InVia) using a 532 nm solid state (100 mW) laser, in back-scattering geometry using a 50 cm^–1^ edge filter. Temperature dependent Raman measurements were carried out using a Linkam (THMS600) Temperature Controlled Stage. Platinum electrodes were coated onto the faces of the sintered pellets for electrical measurements. Capacitance measurements were carried out with a Novocontrol (model Concept 40) measurement system. Polarisation (*P*) and strain (*S*) *versus* electric field (*E*) loops were recorded at 1 Hz from –100 °C to 60 °C using an aixACCT system.

## Results

**Fig. 1:**
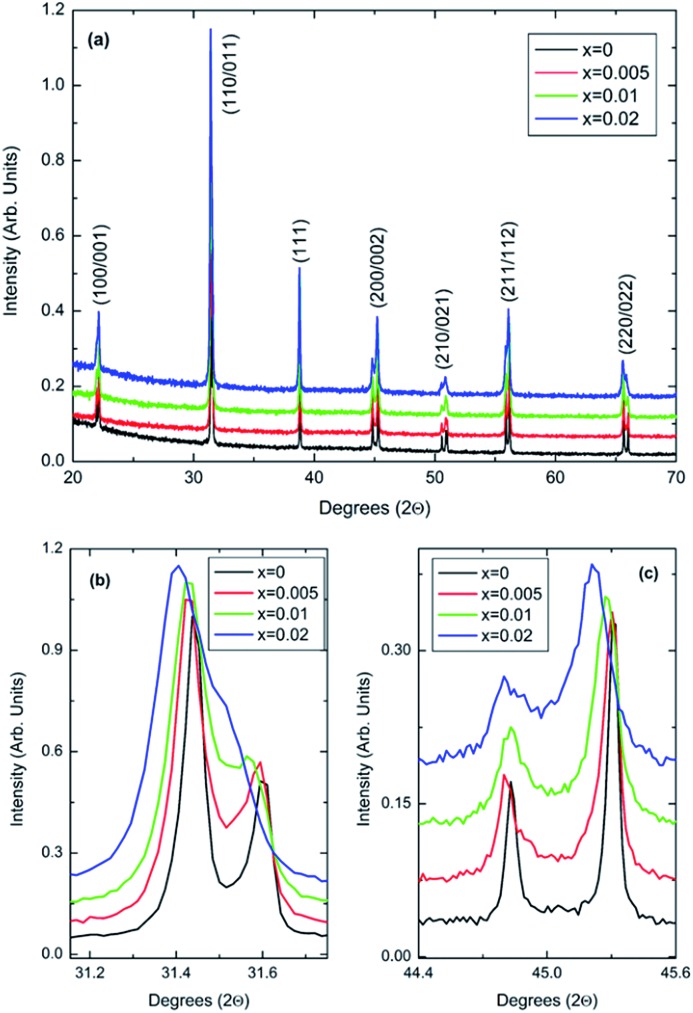
(a–c) Room-temperature XRD data for (1 – *x*)BaTiO_3_–*x*BiYbO_3_ ceramics in the 0 ≤ *x* ≤ 0.02 range.

Room-temperature X-ray data, Fig. 1, shows all (1 – *x*)BaTiO_3_–*x*BiYbO_3_ (BBTYb) ceramics in the 0 ≤ *x* ≤ 0.02 range to be isostructural and their average crystal symmetry to be well described by the non-centrosymmetric tetragonal *P*4*mm* space group. The degree of tetragonality decreases only marginally, as shown by the decrease of the peak splitting in Fig. 1b and (c).

The *P*4*mm* tetragonal structure BaTiO_3_ crystal structure given by R. H. Buttner and E. N. Maslen^16^ was used as a starting model for Rietveld refinements. All of Yb^3+^ was placed in the Ti^4+^ site, whereas Bi^3+^ was placed in the Ba^2+^ site. All datasets were successfully refined with single-phase *P*4*mm* structures. For *x* = 0.02 data O2 temperature factor was fixed at zero. Results are given below. Lattice metrics are listed in Table 1. Refined coordinates, temperature factors (*U*_iso_) and bond lengths are given in Table S1 from the ESI.†

**Table 1:** Lattice metrics for (1 – *x*)BaTiO_3_–*x*BiYbO_3_ ceramics in the 0 ≤ *x* ≤ 0.02 range

*x*	0	0.005	0.01	0.02
*a* (Å)	3.99715(5)	3.99764(7)	3.99923(7)	4.00413(10)
*c* (Å)	4.03660(6)	4.03578(9)	4.03444(9)	4.03492(12)
*V* (Å^3^)	64.4937(14)	64.496(2)	64.526(2)	64.692(3)
*c*/*a*	1.00987(3)	1.00954(4)	1.00880(4)	1.00769(6)

**Fig. 2:**
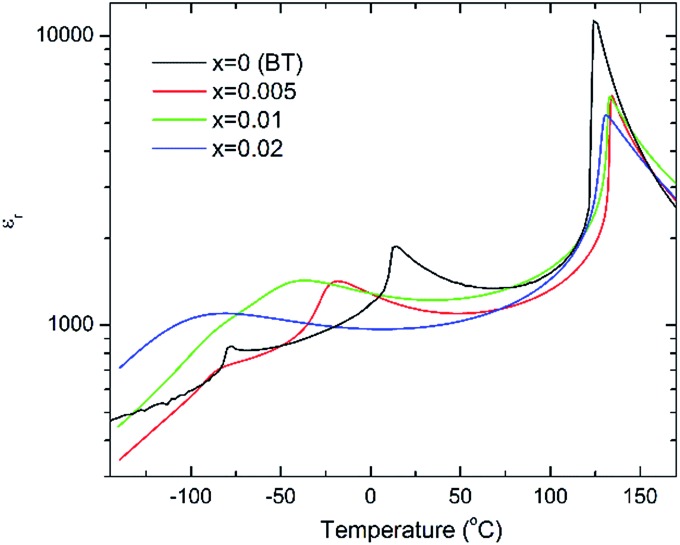
Temperature dependence of the relative permittivity for (1 – *x*)BaTiO_3_–*x*BiYbO_3_ ceramics measured at 10 kHz.

The *c*/*a* ratio is consistent with the aforementioned small decrease in the degree of tetragonality. Also the increase in the unit cell volume with increasing *x* is in agreement with the shift of the diffraction peaks towards lower 2*θ* degrees. On the other hand, the Curie temperatures follow a more irregular trend. First, the Curie temperature, *T*_C_, increases slightly from ∼124 °C for undoped BT to ∼134 °C for *x* = 0.005, as illustrated in Fig. 2. Then it remains almost invariable for *x* = 0.01 and slightly drops to ∼131 °C for *x* = 0.02. This might be ascribed to the combined effect of Bi^3+^ (promoting tetragonality) and Yb^3+^ (promoting pseudocubicity) substitution at A- and B-sites of BaTiO_3_, respectively.^2^,^11^ In contrast, the dielectric anomaly associated with the tetragonal-to-orthorhombic phase transition shifts dramatically towards lower temperatures with increasing Bi/Yb content. Indeed, it drops from ∼12 °C for undoped BT to ∼ –18 °C for *x* = 0.005 and subsequently to ∼–37 °C for *x* = 0.01, eventually merging (at *x* = 0.02) with the orthorhombic-to-rhombohedral phase transition, which remained almost independent of the Bi/Yb content.

**Fig. 3:**
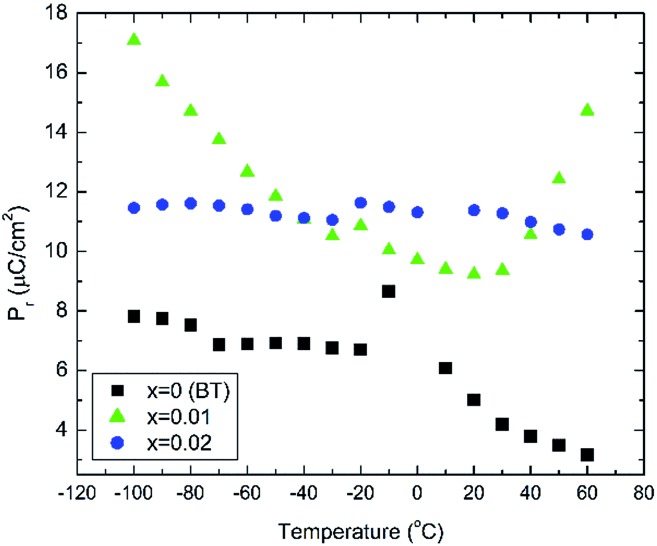
Temperature dependence of the remanent polarisation, *P*_r_, for (1 – *x*)BaTiO_3_–*x*BiYbO_3_ ceramics.

*In situ* electric field-induced polarisation and strain measurements for *x* = 0, 0.01 and 0.02 were carried from –100 °C to 60 °C, under an applied electric field of 20 kV cm^–1^. Temperature dependence of the remanent polarisation, *P*_r_, is illustrated in Fig. 3, whereas temperature dependence of the maximum polarisation, *P*_max_, and the coercive field, *E*_c_, is given in Fig. S2(a, b) of the ESI.† All values have been extracted from the P–E loops presented in Fig. S3 of the ESI.†

Anomalies in *P*_r_ are observed at temperatures similar to those of the dielectric anomalies. These anomalies are more evident in *x* = 0. In addition, *x* = 0.02 ceramics exhibit the most temperature stable *P*_r_, also in broad agreement with the temperature dependence of the relative permittivity shown in Fig. 2. Below –30 °C, BBTYb (*x* = 0.01) exhibits the largest temperature gradient in *P*_r_, however the sudden rise of *P*_r_ above 30 °C, should be regarded with caution, because it may be an artefact caused by the larger leakage current exhibited by this composition, which is also corroborated by the rounder P loops, as shown in Fig. S3 of the ESI.†

**Fig. 4:**
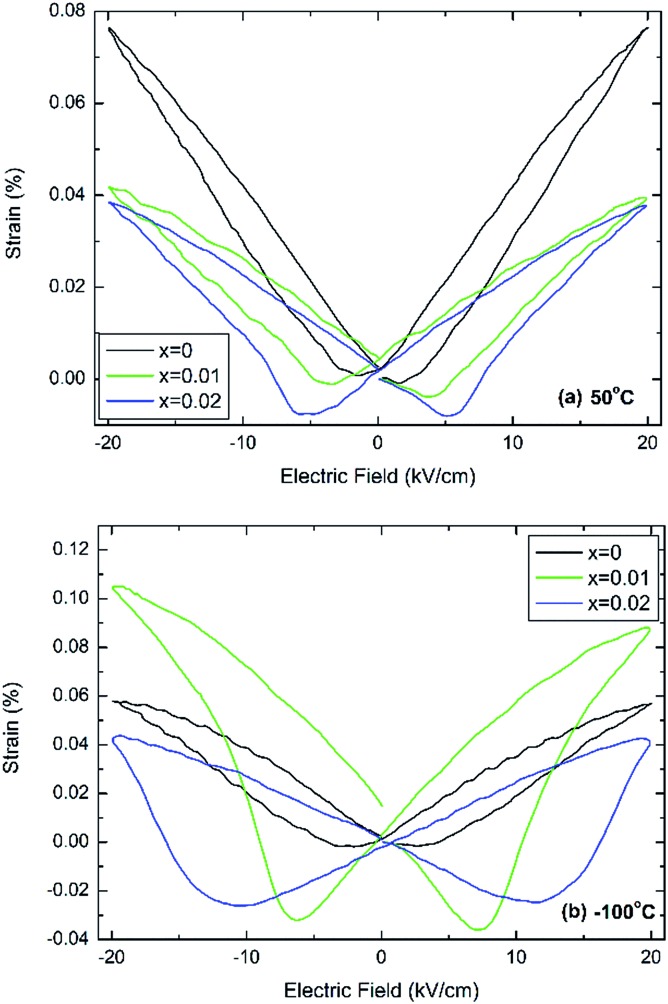
Electric-field induced strain at (a) 50 °C and (b) –100 °C for (1 – *x*)BaTiO_3_–*x*BiYbO_3_ ceramics.

Fig. 4 shows bipolar electric-field induced strain measurements taken at 50 °C and –100 °C, which in case of undoped BT are temperatures well within the tetragonal and rhombohedral phase fields, respectively.

It is clearly evident that this minor doping mechanism also affects dramatically the electromechanical response. In particular, negative strains associated with domain switching increase with increasing *x* and decreasing temperatures. Interestingly, at 50 °C the doped compositions exhibit similar field-induced strains, Fig. 4a, but at –100 °C *x* = 0.01 exhibits a field-induced strain that it is more than the double of the strain shown by 0.02 ceramics, as shown in Fig. 4b. The electric-field associated to the switching of the polarisation parallel to the direction of the electric field increases on cooling, also in agreement with results in Fig. S2b of the ESI,† which appears to increase more dramatically for *x* = 0.02 ceramics.

**Fig. 5:**
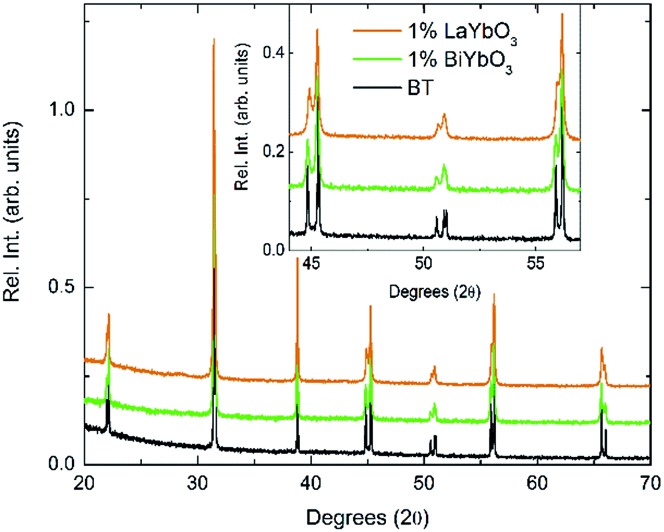
Room-temperature XRD data for (1 – *x*)BaTiO_3_–*x*BiYbO_3_ and (1 – *x*)BaTiO_3_–*x*LaYbO_3_ ceramics in the 0 ≤ *x* ≤ 0.01 range.

In order to rationalise the potential impact of the electronic lone-pair in Bi^3+^ on the dielectric response, (1 – *y*)BaTiO_3_–*y*LaYbO_3_ (BLTYb) ceramics with *y* = 0.01 were prepared. Fig. 5 compares the room-temperature X-ray data for BBTYb ceramics, *x* = 0 and 0.01, and with those of (1 – *y*)BaTiO_3_–*y*LaYbO_3_ (BLTYb) ceramics with *y* = 0.01. The lattice parameters for *y* = 0.01 were calculated as *a* = 3.99801(7) Å and *b* = 4.02584(8) Å leading to a unit cell volume of 64.349(2) Å. The *c*/*a* ratio is 1.00696(4), which is smaller than the value calculated for *x* = 0.01, shown in Table 1.

**Fig. 6:**
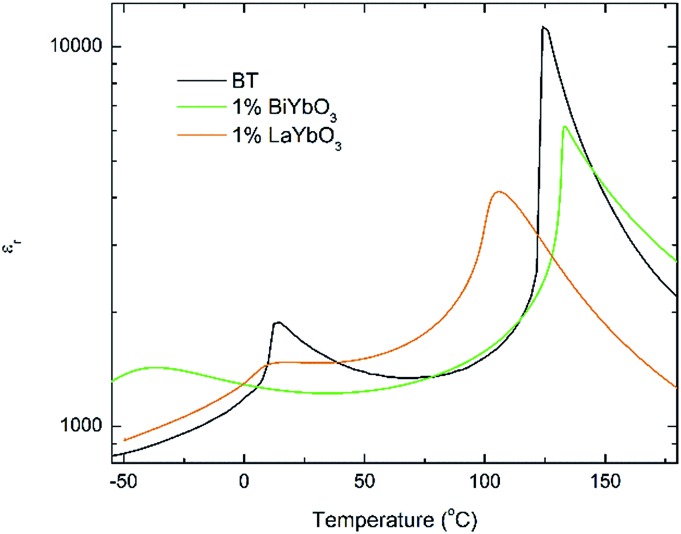
Temperature dependence of the relative permittivity for (1 – *x*)BaTiO_3_–*x*BiYbO_3_ and (1 – *x*)BaTiO_3_–*x*LaYbO_3_ ceramics in the 0 ≤ *x* ≤ 0.01 range.

Again, they all are isostructural and their average crystal symmetry can be described by the non-centrosymmetric tetragonal *P*4*mm* space group. The inset in Fig. 5 shows BLTYb to have a slightly lower degree of tetragonality. Temperature dependence of the relative permittivity of these ceramics is illustrated in Fig. 6. The difference between the dielectric response of the BBTYb (*x* = 0.01) and BLTYb (*y* = 0.01) is remarkable. First, *T*_C_ drops from ∼124 °C for undoped BT to ∼106 °C for BLTYb (*y* = 0.01), whereas it increases to ∼133 °C for BBTYb (*x* = 0.01). From XRD data all ceramics appear isostructural, with identical average structure, therefore the dielectric behaviour may be associated to subtleties on the local structure. Similar subtleties were invoked by Liu *et al.*^17^,^18^ to explain the evolution of the dielectric properties in the K_0.5_Na_0.5_NbO_3_–Bi_0.5_Na_0.5_TiO_3_ system. Those authors used both diffraction and Raman spectroscopy to obtain a better picture of the average *vs.* local structure. Feteira *et al.*^19^ have previously used similar approach to investigate some BaTiO_3_–BiMeO_3_ systems.

Raman spectroscopy is an excellent investigatory technique to evaluate the impact of doping on the local structure, which ultimately may affect the observed temperature dependence of the dielectric response. Indeed, Raman spectroscopy has a shorter length scale than XRD, thereby it allows the identification of local deformations arising from the difference between the ionic radii of Ba^2+^ (1.61 Å), Bi^3+^ (1.45 Å, extrapolated from Shannon), Ti^4+^ (0.605 Å) and Yb^3+^ (0.868 Å). Analysis of the temperature dependence of specific spectral features can be employed to elucidate how the local structure evolves with the temperature and consequently how the structural phase transitions are affected. This approach was employed in past studies carried out in single-crystal, ceramic and thin film specimens. Indeed, the temperature dependence of some transverse optical (TO) modes of *A*_1_ symmetry has been shown to provide sufficient evidence for either presence or absence of the expected structural phase transitions exhibit by BaTiO_3_-based crystals, ceramics and thin films. In addition, again the presence or absence of a low frequency overdamped soft mode provides also a good indication for the type of local symmetry, as discussed later.

**Fig. 7:**
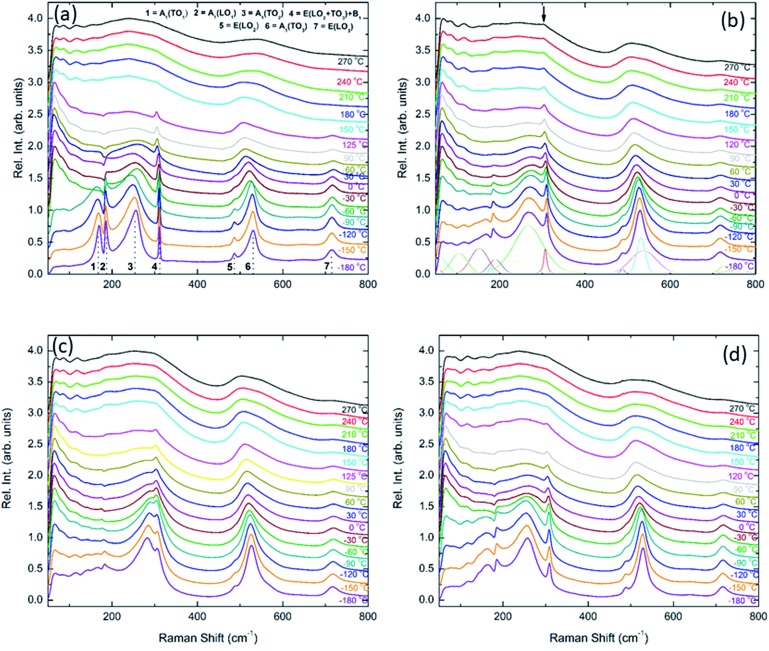
*In situ* Raman spectroscopy for (a) undoped BaTiO_3_, (b) 1 mol% doped BiYbO_3_, (c) 2 mol% doped BiYbO_3_ and (d) 1 mol% doped LaYbO_3_.

Unpolarised temperature dependent Raman spectra for BBTYb *x* = 0, 0.01 and 0.02 and BLTYb *y* = 0.01 ceramics are shown in Fig. 7a–d. Raman spectra for undoped BaTiO_3_ ceramics (Fig. 7a) are in broad agreement with the classical work by Perry and Hall^20^ on single-crystal and with a more recent work by Tenne *et al.*^21^ Moreover, at first glance spectra for doped BBTYb ceramics (Fig. 7) exhibit similar features, where three regions assigned to different vibration modes can be immediately distinguishable. At high temperature macroscopically cubic BT possesses four triply degenerate optical vibrations, namely, 3F_1u_ and 1F_2u_. First-order Raman scattering is symmetry forbidden in paraelectric cubic perovskites, because each ion occupies a center of inversion and all long-wavelength vibrational modes are of odd parity. Nevertheless, non-stoichiometry, inhomogeneous strain distribution, impurities, oxygen vacancies, and dynamic disorder (such as in the paraelectric phase of BT) may break the symmetry and Raman activity can be detected. On cooling, the symmetry of tetragonal BT allows 3(*A*_1_ + *E*) + 1(*B*_1_ + *E*) Raman active optical and 1(*A*_1_ + *E*) acoustic modes.^22^,^23^ Further splitting in *A*_1_ and *E* modes to transverse optic (TO) and longitudinal optic (LO) modes occurs due to the long-range electrostatic force associated with lattice ionicity. In the low- to mid-wavenumber region, the Raman spectrum of tetragonal BT is characterised by a broad *A*_1_(TO_2_) mode at ∼260 cm^–1^ associated with BO_6_ bending vibrations, an interference dip at ∼180 cm^–1^ and a mixed mode *E*(LO_2_ + TO_3_) + *B*_1_ at ∼311 cm^–1^, which only appears in presence of long-range polar order. In particular, the mode at 311 cm^–1^ is related to the cooperative displacement of the central Ti^4+^ with respect to the rest of the unit cell,^23^ and the dip at around 180 cm^–1^ in BaTiO_3_ is attributed to the anharmonic coupling between the three *A*_1_(TO) phonons. Hence, disappearance of the mode at 311 cm^–1^ and of the interference dip at 180 cm^–1^ above 150 °C, is consistent with the tetragonal (ferroelectric) to cubic (paraelectric) phase transition. The *E*(LO_2_ + TO_3_) + *B*_1_ mode at 311 cm^–1^ has been shown to persist at temperatures greater than the permittivity maximum in systems with local polar clusters above *T*_C_.^24^

The aforementioned mode assignment is generally valid only in BT single-crystals. In randomly oriented polycrystalline BT, due to the Raman probe encompassing a finite number of crystallites with various orientations, the directions of the phonon wavevectors are randomly distributed with respect to the crystallographic axes. Consequently, only quasimodes with no true *A*_1_, *B*_1_ or *E* symmetry can be measured.^25^ This is at the basis of the broad spectral signature of polycrystalline BT, and this phenomenon is further complicated in presence of compositional disorder or defects, which cause a relaxation of the Raman selection rules.^26^ Hence, it was chosen in this work to number consecutively the spectral features rather than providing a precise assignment.

The Raman spectrum of undoped BT at –180 °C, well within the single rhombohedral phase field, presents two sharp modes (1, 2) at 169 cm^–1^ and 185 cm^–1^, generally associated with the rhombohedral phase, and a broad mode (3) at 255 cm^–1^. These were assigned by Tenne *et al.*^21^ as *A*_1_(TO_1_), *A*_1_(LO_1_) and *A*_1_(TO_2_), respectively, using single-crystals. The sharp mode (4) at ∼311 cm^–1^ is the one related to ferroelectricity (*i.e.* mixed mode *E*(LO_2_ + TO_3_) + *B*_1_ in single crystals). In the high-wavenumber region there is a weak mode (5) at 486 cm^–1^, plus two broad modes (6) and (7) at 530 cm^–1^ and 714 cm^–1^, respectively. Mode 6 has been assigned by Tenne *et al.*^21^ as *A*_1_(TO_3_). A representative fitting of the investigated spectra is shown at the bottom of Fig. 7b.

**Fig. 8:**
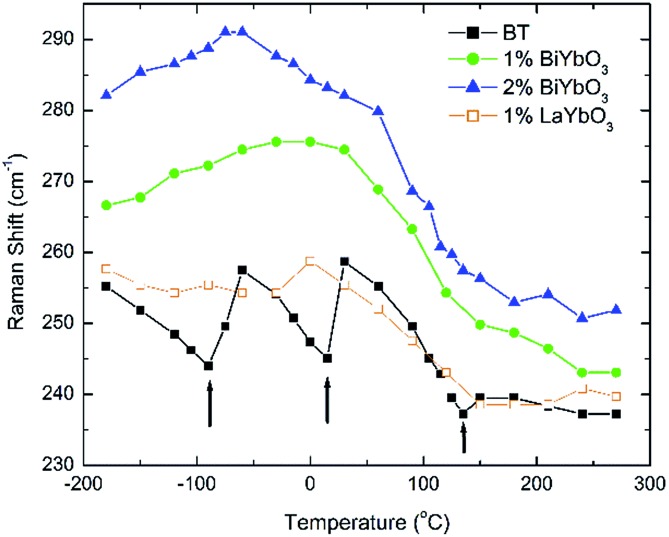
Temperature dependence of the frequency of mode 3 in undoped BT and doped BBTYb and BLTYb ceramics.

Temperature dependent Raman spectra for all compositions are also reported in Fig. 7. The most striking features are the broadening of the FWHM of all modes with increasing temperature, the persistence of mode 4 (at ∼311 cm^–1^) up to high temperature for 1% BiYbO_3_ (*cf.* arrow in Fig. 7b), and the shift of mode 3 (∼255 cm^–1^) with temperature. The first feature is related to the effect of anharmonic terms in the crystal potential energy, whereas the second one will be discussed later. The temperature dependence of the frequency of mode 3 in undoped BT and doped BBTYb and BLTYb ceramics is compared in Fig. 8.

With increasing temperature Raman spectra show large changes, the most noticeable being the sudden hardening of the mode 3 at ∼–80 °C and ∼15 °C, respectively. Those changes are well correlated with the dielectric data shown in Fig. 2, and can be used to monitor the rhombohedral-to-orthorhombic and orthorhombic-to-tetragonal structural phase transitions, respectively. At first instance the absence of sudden Raman shifts for the frequency of mode 3 in BBTYb and BLTYb ceramics could suggest the absence of structural phase transitions, however that would be inconsistent with the dielectric data in Fig. 2 and 6, which show clear anomalies. Hence, a strategy to analyse the temperature dependence of the Raman activity in order to extract the maximum information involves monitoring simultaneously the presence of mode 1 (169 cm^–1^), mode 2 (185 cm^–1^) and of the overdamped soft mode below 100 cm^–1^. The simultaneous occurrence of 1 and 2 provides a strong indication for the presence of regions with rhombohedral symmetry,^26^ whereas identification of tetragonal and orthorhombic symmetries from the spectra is a more delicate procedure, because the overdamped soft mode is detectable in both phases.^27^ Even in single-crystals the difference between these two phases is not pronounced in unpolarized Raman spectra, as shown by Tenne *et al.*^21^ Nevertheless, the overdamped soft mode is not detectable in the rhombohedral phase, as shown in Fig. 7a, in the temperature range –180 °C to –90 °C for undoped BT. Hence, this can be used to ascertain a pure rhombohedral symmetry *versus* mixed crystal symmetries. Let us now inspect the low temperature Raman spectra for doped materials. For example, at BBTYb (*x* = 0.01), as shown in Fig. 7b, modes 1 and 2 coexist with a overdamped mode at low frequencies down to temperatures as low as –180 °C. This suggests the coexistence of rhombohedral and possibly orthorhombic symmetries at low temperatures. In the case of the La-doped counterpart, BLTYb (*y* = 0.01), as shown in Fig. 7d, a similar coexistence is observed only down to –90 °C. In BBTYb (*x* = 0.02), Fig. 7c, modes 1, 2, and overdamped soft mode also coexist down to –180 °C. It is worth mentioning that overdamping is observed also in the orthorhombic and tetragonal phases and it increases with temperature, in agreement with earlier reported results.^21^ Finally, the evolution of the mode 4 at ∼311 cm^–1^ also provides an interesting insight into the polarisation at high temperatures. For undoped BT, this mode disappears in correspondence of *T*_C_, whereas in BBTYb (*x* = 0.01) it persists to temperatures as high as 270 °C, as indicated by the arrow in Fig. 7b. This indicates the persistence of polar clusters with Ti off-centering up to this temperature. With increasing *x*, this mode disappears again nearby *T*_C_, as shown in Fig. 7c. Basically, this mode shows similar behaviour in BT, BBTYb (*x* = 0.02), and BLTYb (*y* = 0.01), Fig. 7a, c, d, respectively.

## Discussion

Room-temperature X-ray diffraction analysis showed all (1 – *x*)BaTiO_3_–*x*BiYbO_3_ (BBTYb) (0 ≤ *x* ≤ 0.02) and (1 – *y*)BaTiO_3_–*y*LaYbO_3_ (BLTYb) (0 ≤ *y* ≤ 0.01) ceramics to be isostructural, and their average crystal symmetry to be described by the non-centrosymmetric tetragonal *P*4*mm* space group, as shown in Fig. 1 and 3. Moreover, as expected the incorporation of Yb^3+^ in the B-site of the BT lattice results in the increase of the lattice cell volume due to its larger ionic radii in comparison with Ti^4+^, as shown in Table 1. Reduction of the degree of tetragonality is also observed in both systems, in particular to a larger extent in the 1 mol% LaYbO_3_ doped ceramics in comparison with their BiYbO_3_ counterparts, as shown in Fig. 3. Nevertheless, the compositional dependence of the structural phase transitions for those two systems is profoundly dissimilar, as shown in Fig. 2 and 6. The impact of substitutional doping in both crystal structure and dielectric properties of BaTiO_3_ has been investigated for more than seventy years. It is often observed that doping affects the temperatures at which the structural phase transitions occur. In most cases, there is a threshold doping level at which the coalescence of those phase transitions also occurs. In the (1 – *x*)BaTiO_3_–*x*BiYbO_3_ that was reported to occur at *x* ∼0.05.

Let us initially focus on the variation of the Curie temperature, which drops from ∼124 °C for undoped BT to ∼106 °C for 1 mol% LaYbO_3_, but increases to ∼133 °C for 1 mol% BiYbO_3_, as shown in Fig. 6 and 2, respectively. Generally, the impact of chemical doping on *T*_C_ is rationalized in terms of simple ion-size effects and/or changes in the tolerance factor, *t*, arguments. For instance, partial replacement of Ba^2+^ (1.61 Å) by the smaller Sr^2+^ reduces the tolerance factor, and *T*_C_ decreases, as the cubic polymorph is stabilized at lower temperature. Nevertheless, exceptions to this empirical rule exist, due to subtleties of the local structure. For example, in the case of Ba^2+^ replacement by Ca^2+^, *T*_C_ initially rises up to 138 °C due to A-cation size variance associated with the substantial size mismatch in the ionic radius of the Ba^2+^ and Ca^2+^ on the A-site, which induces strain in the lattice. Upon further doping *T*_C_ decreases because absolute size effects dominate over size variance. Levin *et al.*^28^ showed both ferroelectric Ca displacements and their amplification of the Ti off-centring to mitigate the lattice-volume effects. Interestingly, in Ca-doped BT with oxygen vacancies for charge compensation, p-type semiconductivity was attributed to the generation of O^–^ ions.^29^

Another exception is the replacement of Ba^2+^ by Pb^2+^, however in this case the increase in *T*_C_ is ascribed to stereological effects associated with the presence of a lone-pair of electrons in Pb^2+^ (6s^2^6p^0^), which leads to the off-centering of Pb^2+^. This can also be empirically explained by a simple mixing rule. Basically, a linear variation of *T*_C_ can be expected between the Curie temperatures for PbTiO_3_ (*T*_C_ ∼ 490 °C) and BaTiO_3_ (*T*_C_ ∼ 130 °C) due to the co-solubility of those two ferroelectric perovskites. Similarly, an increase of *T*_C_ for BaTiO_3_ can be brought by Bi^3+^, which also exhibits a lone-pair of electrons in Bi^3+^ (6s^2^6p^0^). Lattice modifications induced by Bi^3+^ are expected to impact also the shape of the octahedral cage.^30^ These lone-pair effects on the distortion of the BaTiO_3_ lattice were already evidenced by Deluca *et al.*^11^ in the (1 – *x*)BaTiO_3_–*x*Bi_0.5_Na_0.5_TiO_3_ system: using Raman spectroscopy they proved the correspondence between these structural changes and the increase of *T*_C_ with increasing *x*. Similarly, Liu *et al.* suggested that lone-pair effects underpin the evolution of the dielectric properties in the K_0.5_Na_0.5_NbO_3_–Bi_0.5_Na_0.5_TiO_3_ system. Moreover, above some Bi_0.5_Na_0.5_TiO_3_ content, Bi^3+^ will impose octahedral tilting. In the present case, the Bi^3+^ content is in principle rather low to impose any noticeable tilting of octahedra.

Several past investigations showed that La^3+^ doping of BaTiO_3_ according to a Ba_1–*x*_La_*x*_Ti_1–*x*/4_O_3_ mechanism with the creation of B-site vacancies reduces *T*_C_ by 24 °C/at%.^31^ Hence, one can anticipate that the simultaneous incorporation of La^3+^ and Yb^3+^ into BaTiO_3_ would lead to a reduction of *T*_C_, as shown in Fig. 6 and also in ref. 14, for higher doping levels. Basically, the *T*_C_ drops due to a combination of an ion size effect brought by the partial replacement of Ba^2+^ with the smaller La^3+^ and a disruption of the correlated Ti^4+^displacements because of Yb^3+^ incorporation in the B-site. Plausibly, this latest mechanism should also be operating in the (1 – *x*)BaTiO_3_–*x*BiYbO_3_ system, therefore the slight increase of *T*_C_ at low *x* levels, as shown in Fig. 2, needs to arise from the lone-pair effect caused by Bi^3+^. The different behaviour between La^+3^ and Bi^3+^ can be evidenced by plotting the Raman spectra of pure and substituted BT in function of composition.

**Fig. 9:**
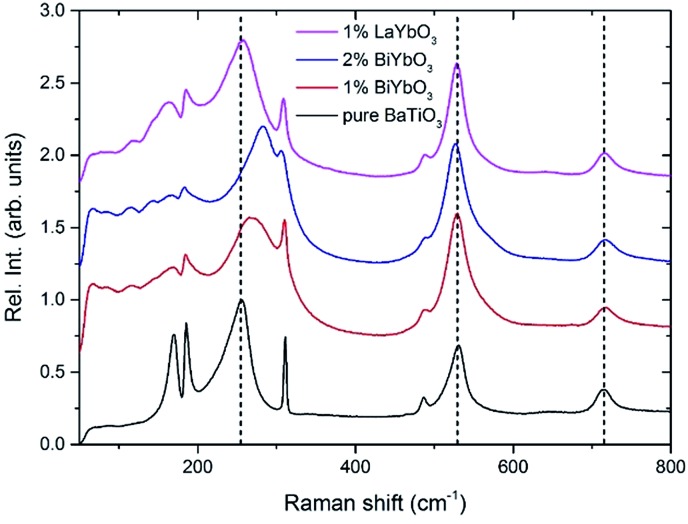
Composition-dependent Raman spectra of BiYbO_3_ and LaYbO_3_-doped barium titanate at –180 °C.

Fig. 9 reports the Raman spectra of BT, BBTYb (*x* = 0.01, 0.02) and BLTYb (*x* = 0.01) at *T* = –180 °C. As could clearly be seen, mode 3 (at 255 cm^–1^) shifts considerably with increasing *x* for the BBTYb compositions, whereas it experiences only a slight shift in BLTYb. This mode is associated to the vibration of the O–Ti–O bond along the polar axis of the unit cell,^11^ hence a shift of this mode is associated with changes in bond length. These can be caused either by the presence of a dopant ion with different mass/size substituting on Ti sites, or by structural distortions in the BaTiO_3_ unit cell. Considering that the composition with 1% LaYbO_3_ displays a negligible shift of mode 3 compared to the one with 1% BiYbO_3_, it seems unlikely that these spectral changes in the BiYbO_3_ compositions are related to Yb^3+^ substitution. Rather, they are associated with the presence of the Bi^3+^ at the A-site, whose lone-pair of electrons promotes Bi off-centring and hybridization with oxygen, thereby distorting the O–Ti–O bond.^32^ The strain induced by Bi^3+^ addition could be also at the basis of the persistence of the mode 4 (at ∼311 cm^–1^) well above the Curie temperature, as determined from the permittivity measurements (*cf.*Fig. 7b and 2). The strain induced by Bi–O bond hybridization could in fact locally stabilize Ti off-centring up to higher temperatures, without the material being macroscopically ferroelectric. This effect is present only for 1% BiYbO_3_; for *x* = 0.02 the higher Yb content, which disrupts Ti off-centring, likely prevents persisting polar clusters at high temperatures.

A further indication of this effect is provided by the shift of mode 3 with increasing *x* in BBTYb. This shift entails an increase of the force constants due the covalent nature of the Bi–O bond.^32^ Since the mode is known to soften with increasing hydrostatic pressure, the measured mode hardening with the incorporation of Bi^3+^ suggests the occurrence of local tensile bond strain. This strain engendered by Bi^3+^ raises *T*_C_, however only at very low doping contents (below 0.02) (when for example compared with Ca (up to 0.08)), because this mechanism is mitigated by the disruption to the Ti-correlated displacements, brought in by Yb^3+^ B-site occupancy.

This can be seen also from the broadening of mode 3 (*cf.*Fig. 9) evident at low (1%) BiYbO_3_ content, which is likely related to strain-induced disorder effects at the perovskite A- and B-sites. The decreased FWHM of mode 3 (at ∼255 cm^–1^) for Bi contents >1% suggests that increased Yb^3+^ addition reduces the disorder due to strain at the B-site. The broadening of mode 1 (∼169 cm^–1^), on the other hand, occurs also for La-doped systems and thus is related only to substitutional disorder (*i.e.* not to strain).

In summary, the lattice disturbance caused by this doping mechanism affects the balance between long-range dipolar coulombic forces and short-range repulsion forces and leads to a larger stability of the tetragonal symmetry (lower polarisability) over both the orthorhombic and rhombohedral (higher polarisability). This is corroborated by the wider temperature range where the tetragonal phase is stable, as illustrated in Fig. 2 for the permittivity behaviour. Here, one should note that the polarization is sustained to a larger temperature because of the influence of Bi^3+^ on the Ti off-centring along the *c*-axis, as suggested by the Raman shift of mode 3 (*cf.*Fig. 7 and 8). These results contradict the current perception, which attributes all of the effect on *T*_C_ to ferroelectric Bi^3+^ displacements. This is strictly valid only in systems that are substituted with Bi^3+^ only on the A-site.^11^ As soon as a large cation like Yb^3+^ is also substituted on the B-site, even a low substituent amount is sufficient to disrupt Ti off-centering and destabilize the local polar regions induced by Bi^3+^, with the consequence that *T*_C_ shifts again to lower temperatures.

It is also known that doping not only affects the Curie temperature, but also the temperatures at which the two other structural phase transitions occur. Hence, let us now consider how these minor doping contents (BBTYB 0.01 and BLTYb 0.01) affect the temperatures of the rhombohedral-to-orthorhombic (*T*_r–o_) and orthorhombic-to-tetragonal (*T*_o–t_) phase transitions. The *T*_o–t_ exhibits a small shift towards lower temperature, from ∼12 °C for undoped BT to ∼10 °C for 1 mol% LaYbO_3_, but a massive drop to ∼–37 °C for 1 mol% BiYbO_3_, as suggested at first glance from the dielectric anomalies shown in Fig. 6. Raman data in Fig. 7 and 8 provide a significant insight into the local crystal symmetries over the temperature range encompassing the sub-ambient structural phase transitions. First, the coexistence of modes 1 and 2 with an overdamped mode at low frequencies, as shown in Fig. 7b–d suggests the coexistence of rhombohedral and possibly orthorhombic symmetries over wider temperatures. Second, softening of the O–Ti–O mode (3) with increasing temperature can be expected from the lattice thermal expansion, which implies a lowering of the force constants. This softening is interrupted at the phase transitions for undoped BT, as indicated by the arrows in Fig. 8. It is worth to note, that it is known that at these phase transitions there is a change of the volume of the unit cell. For all doped compositions the dramatic frequency jump of mode 3 vanishes, despite still noticeable anomalies in the relative permittivity, as shown in Fig. 2 and 6. Interestingly, the diffuse character of those anomalies may arise from internal strains, which origin has already been discussed above. In addition, these strains are also responsible for the coexistence of different local symmetries over wide temperature ranges. Coexistence of the tetragonal and orthorhombic phases is long known in BaTiO_3_.^11^,^33^,^34^ Indeed, in all our compositions (including BLTYb) a peak at ∼190 cm^–1^ is visible in the tetragonal phase, which has previously been assigned to coexistence with the orthorhombic polymorph.^33^ In summary, Bi^3+^ causes local distortions, which lead to the coexistence of crystal symmetries over wide temperature ranges, and for that reason the dielectric anomalies are diffuse in contrast with those observed in undoped BT.

Finally, based on the above analysis we propose an enhanced model for the thermal stability of the permittivity in core–shell type BaTiO_3_ ceramics employed in the manufacturing of MLCCs. It is widely accepted that, in core–shell grains, dopants are present in the shell region, which also shows a compositional gradient, whereas the core region is virtually ferroelectric BT.^35^ The dielectric measurements in Fig. 2 showed how the dependence of the permittivity at low temperatures can be affected by minor amounts of dopants, whereas the Curie temperature remained virtually unaffected. Hence, an imposed compositional gradient from 0 to 2 mol% in the shell region will create a region with relatively stable permittivity in the low temperature regime. Nevertheless, this region has lower capacitance in comparison with the core region consisting of nearly undoped BT. This lower capacitance will cap the maximum permittivity at the Curie temperature for the core region, as those regions can be regarded as two capacitors in series. For details on how an equivalent circuit for a core–shell type microstructure can be evaluated by AC impedance the reader is referred to the classical paper by West *et al.*^36^ and also the recent work by Heath *et al.*^37^

## Conclusions

A complex set of doping mechanisms operate in (1 – *x*)BaTiO_3_–*x*BiYbO_3_ system for low levels of *x*. The simultaneous incorporation of Bi^3+^ and Yb^3+^ into ferroelectric BaTiO_3_ results in the suppression of the low temperature dielectric anomalies. Moreover, Raman spectroscopy analysis provided evidence for a strain mechanism which promotes the coexistence of different crystal symmetries at local level in order to accommodate the local distortions brought in by the presence of a lone-pair of electron in Bi^3+^. This mechanism may be exploited to tune the permittivity in core–shell BT structures and is precursor of the ferroelectric-to-relaxor crossover observed at higher doping concentrations.

## Conflicts of interest

There are no conflicts to declare.

## Supplementary Material

Supplementary information
