# Supplementary material for: Remarkable impact of low BiYbO3 doping levels on the local structure and phase transitions of BaTiO3
Source: J Mater Chem A Mater. 2018 Mar 15;6(13):5443–51. doi: 10.1039/c7ta11096k (PMC5885797; doi:10.1039/c7ta11096k)

Electronic Supplementary Material (ESI) for Journal of Materials Chemistry A.
This journal is © The Royal Society of Chemistry 2018

Electronic Supplementary Information

Remarkable impact of low $BiYbO_3$ doping levels on the local structure and phase transitions of $BaTiO_3$

M. Deluca[a,b], Z.G. Al-Jlaihawi[c], K. Reichmann[d], A.M.T. Bell[c] and A. Feteira[c]

[a]*Materials Center Leoben Forschung GmbH, Leoben, Austria*
[b]*Institut für Struktur- und Funktionskeramik, Montanuniversitaet Leoben, Leoben, Austria*
[c]*Materials and Engineering Research Institute, Sheffield Hallam University, S1 1WB, Sheffield, United Kingdom a.feteira@shu.ac.uk*
[d]*Institute for Chemistry and Technology of Materials, Graz University of Technology Stremayrgasse 9, 8010 Graz, Austria*

Corresponding author: Antonio Feteira, email: a.feteira@shu.ac.uk

Rietveld refinements were done using FULLPROF (Rodríguez-Carvajal, J. (1993). *Phys. B: Condens. Matter*, **192**, 55–69.) The P4mm tetragonal structure $BaTiO_3$ crystal structure (Buttner, R.H. and Maslen, E.N. (1992). *Acta Cryst.* **B48**, 764-769) was used as a starting model for Rietveld refinements.

Table S1. - Refined coordinates, temperature factors (U_{iso}) and bond lengths for (1-x)$BaTiO_3$-x$BiYbO_3$ ($0 \leq x \leq 0.02$) and (1-y)$BaTiO_3$-y$LaYbO_3$ (y=0.01) ceramics.

x	0	0.005	0.01	0.02	0.01 La
Ti/Yb z	0.481(13)	0.472(7)	0.476(7)	0.479(7)	0.488(11)
O1 z	0.04(2)	0.049(14)	0.039(14)	0.038(18)	0.022(20)
O2 z	0.53(2)	0.560(9)	0.551(8)	0.543(8)	0.51(3)
Ba/Bi/La U_{iso} (Å^2)	0.0024(16)	0.0068(14)	0.0037(11)	0.0064(10)	0.0027(8)
Ti/Yb U_{iso} (Å^2)	0.010(4)	0.008(3)	0.004(3)	0.005(2)	0.006(2)
O1 U_{iso} (Å^2)	0.005(15)	0.012(6)	0.004(5)	0.013(11)	0.006(3)
O2 U_{iso} (Å^2)	0.017(10)	0.012(6)	0.004(5)	0	0.006(3)
Ba-O1 x4 (Å)	2.831(5)	2.834(4)	2.832(3)	2.835(4)	2.828(3)
Ba-O2 x4 (Å)	2.76(6)	2.67(2)	2.70(2)	2.72(2)	2.81(8)
Ba-O2 x4 (Å)	2.93(6)	3.02(3)	2.99(2)	2.97(2)	2.87(9)
Ti-O1 (Å)	1.78(10)	1.71(6)	1.76(6)	1.78(8)	1.88(9)
Ti-O1 (Å)	2.26(10)	2.33(6(	2.27(6)	2.26(8)	2.15(9)
Ti-O2 x4 (Å)	2.008(9)	2.030(8)	2.022(6)	2.019(5)	2.001(6)

Fig. S1 Microstructures of $(1\text{-}x)BaTiO_3\text{-}xBiYbO_3$ ($0 \le x \le 0.02$) ceramics.

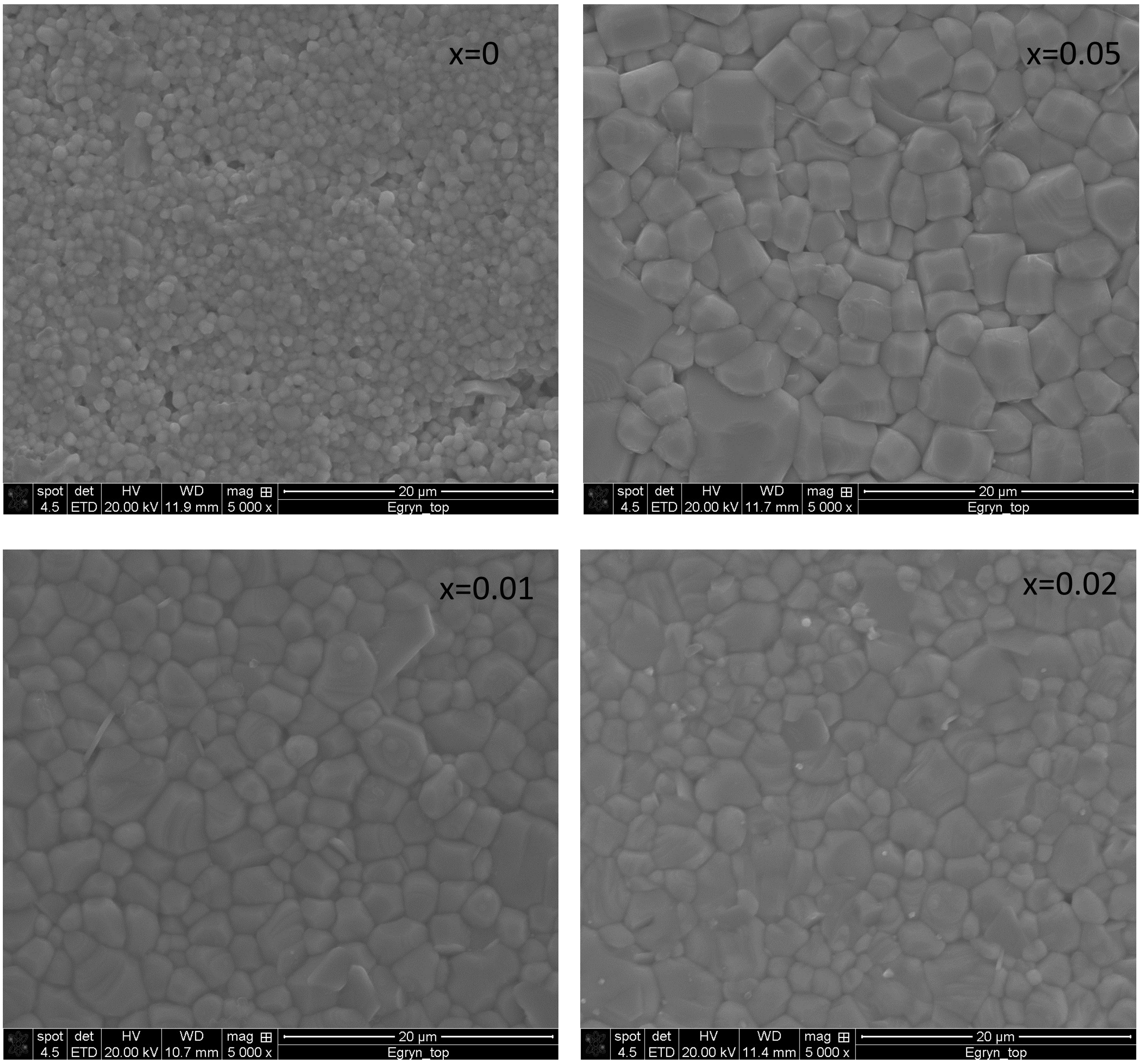

Fig. S2.a Temperature dependence of P_{max} for (1-x)$BaTiO_3$-x$BiYbO_3$ ($0 \leq x \leq 0.02$) ceramics.

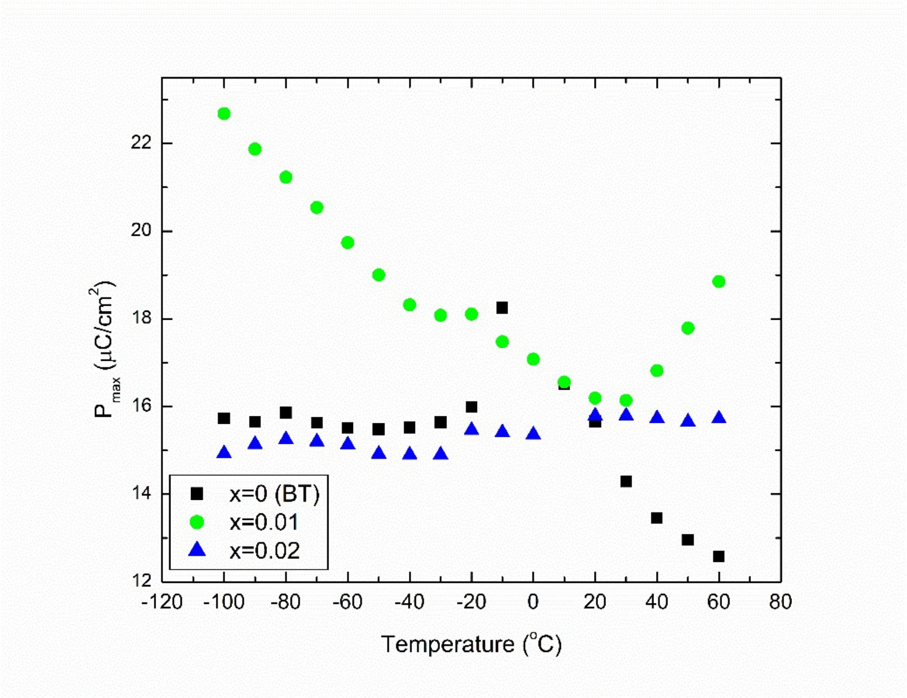

Fig. S2.b Temperature dependence of E_c for (1-x)$BaTiO_3$-x$BiYbO_3$ ($0 \leq x \leq 0.02$) ceramics.

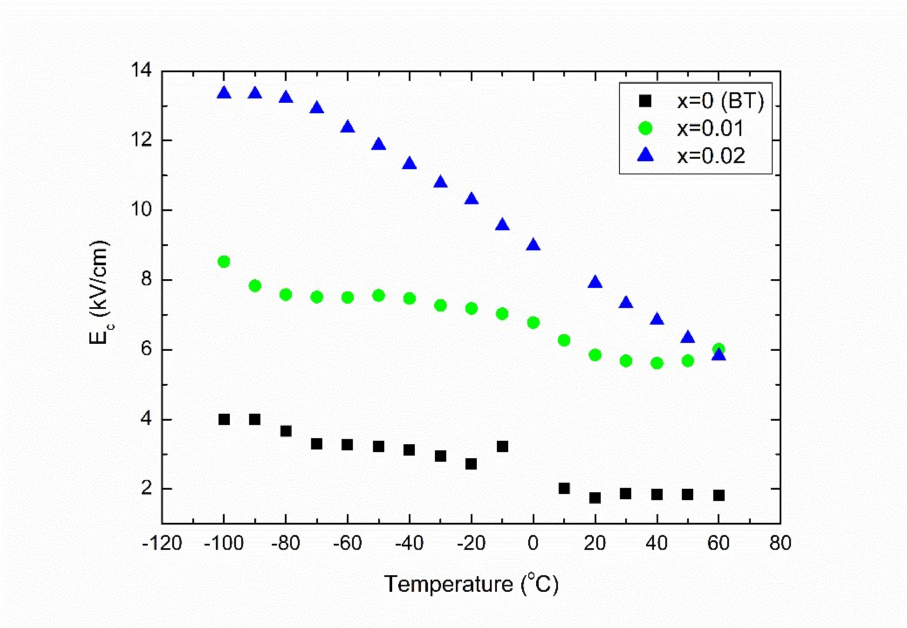

Fig. S3.aTemperature dependence of strain and Polarisation vs electric field for x=0 (BT) ceramics in the temperature range -100 to 50°C.

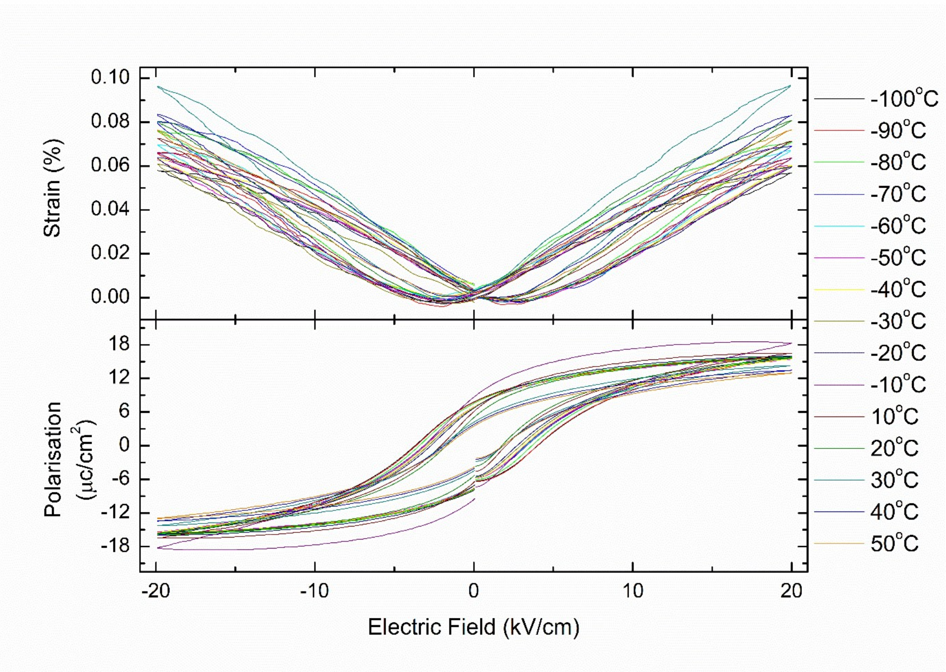

Fig. S3.b Temperature dependence of strain and Polarisation vs electric field for x=0.01 ceramics in the temperature range -100 to 50°C. (arrows indicate decreasing temperatures)

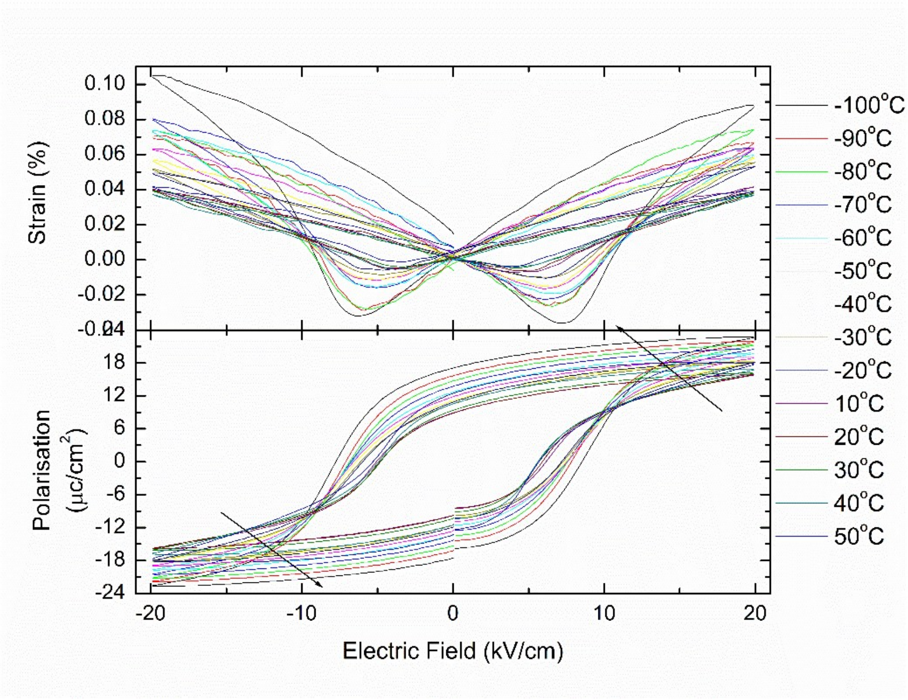

Fig. S3.c Temperature dependence of strain and Polarisation vs electric field for x=0.02 ceramics in the temperature range -100 to 50°C. (arrows indicate decreasing temperatures)

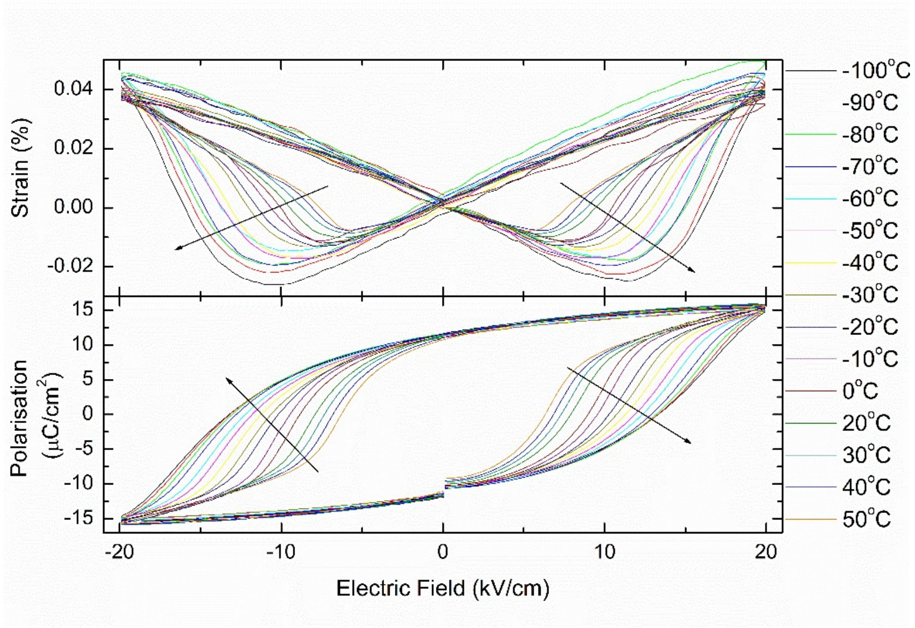

Supplement: Supplementary file 1 [file TA-006-C7TA11096K-s001.pdf]
